# Domiciliary chemotherapy with gemcitabine is safe and acceptable to advanced non-small-cell lung cancer patients: results of a feasibility study

**DOI:** 10.1038/sj.bjc.6601420

**Published:** 2003-12-09

**Authors:** H Anderson, J M Addington-Hall, M D Peake, J McKendrik, K Keane, N Thatcher

**Affiliations:** 1 Department of Medical Oncology, Wythenshawe Hospital, Manchester, UK; 2 Department of Palliative Care and Policy, Guy's, King's and St Thomas' School of Medicine/St Christopher's Hospice, London, UK; 3 Department of Respiratory Medicine, Glenfield General Hospital, Leicester, UK; 4 Eli Lilly and Company Limited, Basingstoke, UK

**Keywords:** domiciliary, gemcitabine, advanced NSCLC

## Abstract

A study was conducted to investigate the feasibility and acceptability of administering single-agent gemcitabine to patients with advanced non-small-cell lung cancer (NSCLC) in their own homes. Gemcitabine is an active agent in NSCLC with a good toxicity profile and lends itself to this type of investigation. A total of 24 patients were studied; as only one patient required gemcitabine to be changed from home administration to hospital administration, domiciliary gemcitabine is feasible. A total of 249 injections of gemcitabine were given, the mean number of courses being 3.5, range 1–6. The gemcitabine was given at 1000 mg m^−2^ on days 1, 8 and 15, the courses being repeated every 28 days. All patients received their first course in hospital and in total 147 were given at home and only 14 in hospital on courses 2–6. Furthermore, both the patients and carers reported positively on the use of domiciliary gemcitabine and preferred it over hospital administration. There was no evidence of increasing burden to community services during the domiciliary chemotherapy. Further studies investigating this approach are warranted.

Patients with advanced non-small-cell lung cancer (NSCLC) treated with cisplatin chemotherapy have a statistically significant increased survival compared with best supportive care alone (NSCLC Collaborative Group, 1995; Cullen *et al*, 1999). The survival benefit has been confirmed on a number of subsequent randomised trials comparing the newer agents (vinorelbine, paclitaxel, docetaxel) against best supportive care alone (Elvis, 1999; Ranson *et al*, 2000; Roszkowski *et al*, 2000). Furthermore, a gemcitabine study demonstrated significant improvements in relief of disease-related symptoms and quality of life (QoL) assessed by an EORTC validated questionnaire, with a reduction in hospitalisation and need for radiotherapy (Anderson *et al*, 2000).

Surveys have been conducted to address the concern about the balance between what was perceived as being toxic therapy in a palliative care setting *vs* the now known benefits of palliative chemotherapy. When patients are asked about their views, they are much more willing to accept chemotherapy than either healthy individuals or health-care professionals (Slevin *et al*, 1990). Furthermore, a study from the United States indicated that patients valued relief of symptoms even though survival gain may be short (Silvestri *et al*, 1998).

Gemcitabine has been evaluated in several trials in NSCLC and has shown independent validated response rates of 18–26%, with median survivals ranging from 6 to 12 months (Noble and Goa, 1997). Gemcitabine also improves disease-related symptoms in more patients than those who obtained an objective tumour response (Thatcher *et al*, 1995a, 1995b, 1997). Gemcitabine has little toxicity with minimal myelosuppression and alopecia (Aapro *et al*, 1998). The agent is relatively easy to give on an outpatient basis and it was therefore considered suitable to evaluate in the domiciliary setting. Perhaps surprisingly, no comparable bolus chemotherapy study appears to have been reported. Domiciliary administration could reduce patient waiting times in the hospital and increase the time spent at home, which is known to be of importance to patients and their families (Grande *et al*, 1998). However, the possible disadvantages of home-based chemotherapy include missing the support of other patients in a similar position, perhaps feeling less secure at home and relatives finding the burden of caring for the patient receiving home chemotherapy too great.

The prime objective of this study was to determine the feasibility of administering gemcitabine in a domiciliary setting to patients with locally advanced or metastatic NSCLC. The impact on the patients' QoL was also measured and an attempt was made to try and determine the acceptability based on patient and carer views.

## PATIENTS AND METHODS

A total of 24 patients with advanced, inoperable NSCLC were entered into the study over 14 months. The protocol entry criteria included no prior chemotherapy and eligibility required bulky stage III (unsuitable for a resection or radiotherapy with curative intent) or stage IV disease, histologically proven NSCLC with a performance status of 0–2. Patients with active infection, brain metastases, hypercalcaemia, a serum creatinine of more than 150 *μ*mol l^−1^, bilirubin more than 1.5 times normal or transaminases more than three times the upper limit of normal were excluded from the study. Patients were accepted if single-agent gemcitabine was considered to be a reasonable alternative given patient factors that were likely to compromise combination chemotherapy, for example, cardiorespiratory comorbidity, etc. Written informed consent was obtained and all patients were analysed.

### Study drug administration

Gemcitabine was administered as an intravenous infusion over 30 min at a dose of 1000 mg m^−2^. Treatment was repeated weekly for 3 weeks followed by a 1-week break. Cycles were repeated every 4 weeks for a maximum of six cycles. Patients' disease-related symptoms were assessed every 4 weeks. Toxicity and response rates were assessed according to standard WHO criteria.

The first course of chemotherapy was administered in the hospital. Prior to the second course of treatment, patients were seen in the outpatient clinic, and blood taken for a full blood count and biochemistry profile. If toxicity following the first course was acceptable, the patient had the second and subsequent cycles of chemotherapy at home. A specialist nurse collected the chemotherapy from the hospital and administered it in the patients' home. Patients were encouraged to telephone the in-patient ward for advice and also had the telephone number of the specialist chemotherapy nurse. A full blood count was taken at each visit, days 8, 15 and 28 by the specialist nurse and used to dose the next chemotherapy treatment. Toxicity and dose modifications were defined by the same protocol whether in-patient or outpatient. If grade 3/4 toxicity was present at the time of planned drug administration, the dose was omitted. If gemcitabine was not given for 6 weeks, then it was discontinued.

### Primary end point

The primary end point was the feasibility of administering gemcitabine in a domiciliary setting based on three components:

- Practicability – if the number of patients requesting reversion to hospital care was less than 25%, then the approach was deemed practical.
- Summaries of dose omission and patient discontinuations.
- Acceptability by assessing toxicity, QoL for patients and carers.

#### QoL assessments

No more than 1 week before the first cycle of chemotherapy, the patients' QoL was assessed using the EORTC-QLQ-C30 questionnaire with the LC13 module for lung cancer. Patients also completed the Hospital Anxiety and Depression scale (HADs). These measures were also completed at the end of the second cycle, that is, the first cycle to be administered at home.

Patients were asked to nominate a family member or friend who was most involved in helping to support them at home. At the same three points during the study, the nominated carer was asked to complete three questionnaires: the Nottingham Health Profile (Hunt *et al*, 1993), an extensively validated, concise measure of subjective health status; the GHQ-28 (Goldberg and Hillier, 1979), a widely used measure of psychiatric disturbance and the Caregiver Strain Index (Robinson, 1983), a measure of the impact of caring.

In addition to the QoL measure, both patients and carers were to be approached for interview by an independent, experienced research interviewer at the end of the second cycle of chemotherapy. The interviews were to be semistructured and explored the respondent's experiences of receiving chemotherapy at home rather than in the hospital. The responses to open questions were recorded verbatim by the interviewer.

### Secondary end points

#### Safety

Clinical laboratory tests were carried out no more than 2 weeks before the patient enrolled into the study and during the study. Changes in relevant laboratory and nonlaboratory values were noted during the study, toxicity was rated using the WHO scale at the end of each cycle and any adverse events reported.

#### Tumour response

No more than 3 weeks before enrolling into the study, the disease status of each patient was assessed. Efficacy was examined during the study at day 21 of each therapy cycle or in the rest week to assess tumour response.

#### Resource utilisation

A resource use analysis was performed on all patients enrolled into the study. Data were collected for each patient at the end of each cycle of chemotherapy. The data collection period was from the date the patient entered the study until 4 weeks after they received their final dose of gemcitabine. For each patient, data were collected as follows:

Distances travelled

- By the patient (between their home and the hospital).
- By the domiciliary nurse between their base and the patient's home.
- Number of visits to the patient's home to administer the gemcitabine and the length of each of those visits.

Number and length of visits

- To the patients home by health-care professionals to deal with adverse events or for any other reason.
- The number of admissions to hospital and the length of stay.
- The additional number of hours taken off work by the carer due to the domiciliary administration of gemcitabine.

### Statistical methods

#### Patient population, drug administration and feasibility

The baseline patient characteristics, details of chemotherapy administration (number of doses, omissions, etc.) and feasibility were summarised.

#### QoL/carer data

Patient EORTC-QLQ-C30, LC13 and HAD scores at baseline were compared to those after the second cycle of chemotherapy. The differences between scores at baseline and cycle 2 were not normally distributed: the Wilcoxon signed-rank test was therefore used to test the statistical significance of these differences.

The recommended weightings were used for only two of the Nottingham Health Profile scales; the vast majority of scores on the remaining scales were zero and McNemar's test of paired proportions was therefore used to detect differences in the proportion of scores greater than zero between the two time periods.

Responses to open questions on the patient and family interviews were grouped into categories.

#### Safety and tumour response

The incidence of toxicity grades was summarised for the study population. The reasons for doses of gemcitabine being omitted were also recorded. Physician-assessed disease-related symptoms, tumour response and chemotherapy toxicity were summarised.

#### Resource utilisation data

The distances travelled by the patient and the domiciliary nurse were summarised for the study group. The number and length of domiciliary nurse visits for gemcitabine administration were reported for each cycle. In addition, the average number of visits per patient by the other types of health-care professionals (GP, District Nurse, Domiciliary Nurse, Other) were summarised for each cycle. The number of patients who had an in-patient stay during the study was noted and the average length of stay was calculated. Although information was to be collected on carer burden, unfortunately, there was only information completed for five patient's carers and the data were not comprehensive enough to allow any summaries to be made.

## RESULTS

### Patient population

The patient characteristics are shown in Table 1

**Table 1:** Patient characteristics

Total	24
Males	15
Females	9
Median age (range)	62.6 years (45–79)

*Zubrod performance status*	
0	3 (12%)
1	12 (50%)
2	9 (38%)

*TNM stage*	
II	1 (4%)
IIIa	1 (4%)
IIIb	10 (42%)
IV	12 (50%)

*Histology*	
Squamous	14 (58%)
Adenocarcinoma	6 (25%)
Undifferentiated	4 (17%)

. All patients were Caucasian and the mean weight was 66 kg (range 41–95). The majority of patients had Zubrod performance status 1 (ambulatory with symptoms) with 10 (42%) stage IIIB patients and 12 (50%) stage IV patients. The patient with stage II tumour had a spiculated 3 cm mass in the right upper lobe with a 6 cm right hilar mass and a subcarinal lymph node of uncertain significance with interstitial lung disease where a definite diagnosis of lymphangitis could not be excluded. This patient and the stage IIIA patient had been reviewed by radiation oncologists and a surgeon for radical treatment, but this was not deemed possible. Three patients had received prior radiotherapy, to lymph node masses (2) and the other to a lung mass, which was not in the site of the only measurable disease.

### Study drug administration

The median number of cycles was two, the mean 3.5 and the range 1–6. A total of 249 injections of gemcitabine were given. No treatments were reduced. Six doses were delayed (three due to holidays, one due to chest infection and two due to nausea and vomiting).

### Primary objective

#### Feasibility

One patient was very anxious and fainted with the day 15 injection of the second cycle of gemcitabine, and all subsequent gemcitabine was given in the hospital for this patient. It was therefore considered that domiciliary administration was feasible by the predetermined criterion as only one out of 24 (4%) patients required hospital care for gemcitabine administration.

#### Dose omissions and patient discontinuations

All patients received their first cycle of chemotherapy in the hospital. Of the 72 planned infusions of gemcitabine in the first cycle, four were omitted (ankle oedema, chest infection, hypertension and flu-like symptoms). According to protocol, all patients who tolerated gemcitabine in the hospital should have their second course of chemotherapy at home. Three patients did not have their domiciliary second cycle as they had withdrawn from the study by this point due to lack of efficacy of gemcitabine (2), and one due to flu-like side effects.

In the second cycle, five of the planned infusions were omitted, four due to adverse events, mucositis, oedema, vomiting on two occasions and one because the patient declined. Five doses of gemcitabine were administered in the hospital during the second cycle due to sore eyes, nausea, syncope and anxiety. The other patient was seen in the hospital to assess pain, received the gemcitabine in the hospital but had ongoing symptoms, so that further gemcitabine was administered in the hospital, whereas the fifth and sixth courses of gemcitabine were given at home. The other four patients were assessed in the hospital and gemcitabine was administered at the hospital visit, but all had their next gemcitabine therapy at home.

Table 2

**Table 2:** Gemcitabine administration

				**Number of infusions**	
**Cycle**	**No. of patients**	**No. of planned infusions**	**Number omitted**	**Home**	**Hospital**
1	24	72	4	—	68
2	21	63	5	53 (91%)	5 (9%)
3	15	45	5	35 (88%)	5 (12.5%)
4	12	36	2	31 (91%)	3 (9%)
5	6	18	2	16 (100%)	0
6	5	15	2	12 (92%)	1 (8%)

shows the proportion of planned gemcitabine treatments given at home and in the hospital and the number of doses omitted for each cycle. Five patients completed all six cycles. Six patients received blood transfusions (a total of 14 units). In all, 20 patients discontinued chemotherapy; five of these were due to adverse events vomiting (course 3), hypertension (course 2), flu-like symptoms (course 1) and dyspnoea (courses 2 and 4). One patient died of a cerebral vascular accident and six patients with lung cancer. The remaining seven were withdrawn due to perceived lack of efficacy and one patient declined further treatment.

Of 147 domiciliary treatments only 14 were given in hospital. A total of 20 planned doses were omitted, 11 because of toxicity (oedema 2, infection 3, malaise 1, hypertension 1, vomiting 2, mucositis 1, coryza 1) and nine because of the patient's wish to stop treatment at the time of day 15 infusion of gemcitabine during the second, fifth and sixth cycles of therapy.

#### Acceptability – patient and carer QoL

Of the 21 patients who completed the second cycle of chemotherapy – the first being administered at home – 16 provided QoL data. The median age of those providing QoL data was 61.5 years compared to 62 years in those who did not.

Scores on the role-functioning scale of the EORTC-QLQ-C30 declined significantly between the baseline and the end of the second cycle of gemcitabine, but the other parameters did not change significantly although there was a trend for worsening appetite and constipation (Table 3

**Table 3:** Comparison of quality-of-life (QoL) scores, as measured by EORTC-QLQ-C30, before the first cycle of gemcitabine and after the second cycle (the first to be administered at home) (*N*=16)

	**Baseline: mean**	**Cycle 2: mean**	**Mean difference**	**Wilcoxon *P***
*Functional scales*^a^				
Physical functioning	60.9	52.5	8.44	0.65
Role functioning	57.3	38.5	18.8	0.01
Emotional functioning	65.4	61.8	3.68	0.22
Cognitive functioning	87.1	74.9	12.2	0.10
Social functioning	71.8	55.2	16.6	0.066
Global health status/QoL (QL2)^a^	62.8	52.9	9.93	0.062
*Symptom scales/items*^b^				
Fatigue	32.3	49.3	−16.9	0.02
Nausea and vomiting	14.6	32.2	−17.7	0.02
Pain	17.6	23.9	−6.31	0.61
Dyspnoea	49.7	49.7	0.00	0.81
Insomnia	28.9	24.8	4.12	0.75
Appetite loss	28.9	51.9	−23.0	0.08
Constipation	16.6	37.2	−20.7	0.053
Diarrhoea	0	6.6	−6.60	0.08
Financial difficulties	22.7	16.5	6.25	0.16

a A high score for a functionalscale or for the global QoL scale represents a high level of functioning/QoL.

b A high score for a symptom scale/item represents a high level of symptomatology/problems.

).

The fatigue and nausea/vomiting scales showed statistically significant increases, but the other items including financial difficulties were not significantly altered. Alopecia on the EORTC Lung Cancer Module was significantly higher at the end of cycle 2 than at baseline, while cough was significantly improved (Table 4

**Table 4:** Comparison of quality-of-life (QoL) scores, as measured by EORTC Lung Cancer Module (LC13), before the first cycle of gemcitabine and after the second cycle (the first to be administered at home) (*N*=16)

	***N***	**Baseline: mean**	**Cycle 2: mean**	**Mean difference**	**Wilcoxon *P***
Dyspnoea	15	31.5	36.7	−5.13	0.39
Coughing	16	43.3	26.8	16.5	0.021
Haemoptysis	16	8.25	0.00	8.3	0.10
Sore mouth	16	8.25	12.4	−4.18	0.52
Dysphagia	16	8.25	10.31	−2.06	0.74
Peripheral neuropathy	15	15.4	13.3	2.13	1.00
Alopecia	15	0.00	13.2	−13.2	0.03
Pain in chest	16	16.5	8.3	8.25	0.10
Pain in arm or shoulder	15	11.00	13.2	−2.2	0.70
Pain in other parts	15	13.2	24.3	−11.1	0.096

A high score represents a high level of symptomatology/problems.

). Changes in haemoptysis, pain in the chest and pain in other parts approached statistical significance. The levels of anxiety and depression as measured by the HADs scale did not increase significantly between baseline and the end of cycle 2 (Table 5

**Table 5:** Comparison of depression and anxiety scores, as measured by the HADs, before the first cycle of gemcitabine and after the second cycle (the first to be administered at home) (*N*=16)

	***N***	**Baseline: mean**	**Cycle 2: mean**	**Mean difference**	**Wilcoxon *P***
Depression	16	4.87	7.25	−1.37	0.055
Anxiety	16	7.19	8.56	−2.37	0.14

A high score represents a high level of symptomatology/problems.

HADs=Hospital Anxiety and Depression scale.

). There were no statistically significant differences between the baseline and the end of cycle 2 in the responses of informal carers on the Nottingham Health Profile, GHQ-28 or Carer Strain Index (Table 6

**Table 6:** Impact on informal carers: comparison of their health status, psychological morbidity and carer strain, before the first cycle of gemcitabine and after the second cycle (the first to be administered at home) (*N*=16)

					**McNemar's test**		
	***N***	**Baseline: mean**	**Cycle 2: mean**	**Mean difference**	**0 at cycle 2 (n)**	**1+ at cycle 2 (n)**	**P**
**Nottingham Health Profile**							
*Energy*^a^							
0 at baseline					11	3	0.62
1/2/3 at baseline					1	1	
*Pain*							
0 at baseline					12	2	0.50
1/2/3/4/5 at baseline					0	2	
Sleep	16	21.2	27.3	−6.13			0.29
Emotional reactions	16	16.5	14.6	1.93			1.00
*Social isolation*							
0 at baseline					9	4	0.38
1 at baseline					1	2	
*Physical mobility*							
0 at baseline					13	2	0.50
1 at baseline					0	1	
GHQ 28							
Total	17	4.76	3.53	1.23			0.21
Caregiver strain index	17	11.11	11.76	−0.65			0.63

A high score represents a high level of symptomatology/problems.

).

Of the 13 patients who were interviewed at the end of cycle 2, 11 thought that having chemotherapy at home had advantages over having it in the hospital, and all 13 thought it had no disadvantages compared with having it in the hospital. All preferred having it at home, and would recommend it to others in the same situation. Two patients reported feeling more secure when having their chemotherapy at home, one that they felt more secure in the hospital and the remaining patients felt as secure at home as in the hospital. Comments made by patients included that that they felt more relaxed at home and found it less stressful (eight patients), that home was better because there was no travelling involved (6), that the hospital was worse because it took time to travel there and to wait for treatment (6), that they preferred to be at home because they could do what they wanted (including having a cup of tea, going to bed immediately after treatment, having a cigarette) (4), that it was better being in their own surroundings (3), that they got better care at home (3) and that it was better because it did not break up their routine (3).

Informal carers were also positive about home administration of gemcitabine. All but one of those interviewed at the end of cycle 2 (17/18) thought that having it at home had advantages for the family over having it in the hospital. Only two reported that it had any disadvantages for the family: one felt very anxious about what to do if there were problems, and the other thought that the side effects of treatment might have been less in the hospital. All but one preferred it when the patient had their chemotherapy at home, and all but two would recommend it to other families in the same situation (the two exceptions were unsure whether they would recommend it). Comments made by informal carers included that having treatment in the hospital involved a great deal of travelling and this was avoided at home (12), that it took less time at home and that there were no waiting times as in the hospital (11), that home administration was better for the patient (9), that it was more relaxing and less stressful to have treatment at home (7), they preferred it because the patients were able to do what they wanted, such as going to bed after treatment (5). Two carers mentioned that it was more convenient for them if the patient had treatment at home.

The patient's reports gave a mean of 1.15 h from the time the nurse arrived to give chemotherapy at home until the nurse left. In contrast, the patients' visit to hospital for chemotherapy had taken a mean of 7.04 h from the time they left home until they got home again; and the mean duration of the hospital visit was 5.73 h.

### Secondary objectives

#### Toxicity and tumour response

Four patients had a partial response (17%), 15 stable disease (63%) with progression in the remainder. No grade IV toxicities were observed for any parameter. However, grade III toxicities occurred for example nausea, vomiting, neutropenia, haematuria and lethargy – see Table 7

**Table 7:** Worst WHO toxicity grades by patient for laboratory parameters

	**Grade 0 (%)**	**Grade 1 (%)**	**Grade 2 (%)**	**Grade 3 (%)**	**Grade 4 (%)**
Haemoglobin	10 (42)	10 (42)	3 (12.5)	1 (4)	0
WBC	14 (58)	5 (21)	3 (12.5)	2 (8)	0
Neutrophils	15 (63)	5 (21)	4 (16)	0	0
Platelets	21 (88)	1 (4)	1 (4)	1 (4)	0
AST/ALT	11 (46)	8 (33.5)	3 (12.5)	2 (8)	0
Alkaline phosphatase	17 (71)	4 (16)	2 (8)	1 (4)	0
Proteinuria	11 (46)	9 (38)	4 (16)	0	0

for the laboratory-based toxicity gradings and Table 8

**Table 8:** Worst WHO toxicity grades by patient for nonlaboratory data

	**Grade 0 (%)**	**Grade 1 (%)**	**Grade 2 (%)**	**Grade 3 (%)**	**Grade 4 (%)**
Haematuria	14 (58)	8 (33)	1 (4)	1 (4)	0
Haemorrhage	24 (100)	0	0	0	0
Oral	14 (58)	9 (37.5)	1 (4)	0	0
Nausea/vomiting	5 (21)	8 (33)	8 (33)	3 (12.5)	0
Diarrhoea	19 (79)	5 (21)	0	0	0
Pulmonary	23 (96)	0	1 (4)	0	0
Fever	20 (83)	4 (17)	0	0	0
Cutaneous	19 (79)	4 (17)	1 (4)	0	0
Alopecia	8 (33)	13 (54)	3 (12.5)	0	0
Infection	21 (88)	2 (8)	1 (4)	0	0
Lethargy	3 (12)	12 (50)	5 (21)	4 (17)	0
Peripheral	19 (79)	5 (21)	0	0	0
Constipation	14 (58)	9 (38)	1 (4)	0	0

for nonlaboratory toxicities.

#### Resource utilisation

##### Distances travelled

The distances travelled by the patients from their home to the hospital ranged from 2 to 83 miles. The average distance was 29 miles (standard deviation=28) and the median was 15 miles. The distances travelled by the domiciliary nurse were greater, ranging from 4 to 113 miles (mean=51, standard deviation=29, median=52). These distances did not include the nurse travelling to the hospital to pick up the chemotherapy.

##### Domiciliary visits for gemcitabine administration

A total of 147 doses of gemcitabine were administered in the patients' home and domiciliary nurses made a total of 150 visits for this purpose. The average length of time spent in the patients' home was 80 min and this average was similar for each of cycles 2–6 individually.

##### Domiciliary visits not for gemcitabine administration

Table 9

**Table 9:** Visits not for administration of gemcitabine

	**No. of patients**		**GP**		**Domiciliary nurse**	**District nurse**		**Other**
**Cycle**	**In cycle**	**With data**	**AE**	**Other**	**Other**	**AE**	**Other**	**Other**
1	24	24	0	8	1	0	17	4
2	21	21	2	10	2	0	28	3
3	15	13	2	1	0	2	16	1
4	12	11	1	1	2	0	7	0
5	6	6	1	0	0	1	1	0
6	5	5	0	0	1	1	1	0

AE=adverse events.

shows the average number of domiciliary visits which each patient had during each cycle. The average number of visits did not seem to increase substantially throughout the duration of the study, although the burden on the GP and district nurse was slightly increased in cycle 2.

##### Hospitalisation

In all, 13 (54%) of the 24 study patients had an in-patient stay during the study period, but no patient had more than one in-patient stay. All the 13 hospitalisations were due to adverse events (DVT, cerebral metastases, intermittent vomiting, anaemia (2), cellulitis, dyspnoea (2), chest infection, vomiting, hypercalcaemia and confusion. The average length of stay was 5.25 days.

## DISCUSSION

At the time of the study (1995), there were no data on the added benefit of combining gemcitabine with platinum. The study did allow better performance patients to enter; however, there were comorbidities, for example, myocardial infarcts, pancreatitis, chronic hearing difficulty, single kidney and patient preference for a possible domicillary regimen. Furthermore, 58% of the patients were receiving opiate-type medication on entry. As reported in previous studies, gemcitabine administration was associated with a reduction in some of the disease-related symptoms (Thatcher *et al*, 1997). There was a statistically significant reduction in patients' cough between study entry and the end of the second cycle of gemcitabine: reductions in haemoptysis and pain in the chest approached statistical significance. In contrast, alopecia, fatigue, nausea and vomiting increased during the study, while there was a trend of appetite loss, constipation and ‘pain in other places' to increase. The mild flu-like symptoms were not measured specifically by the QoL measures used in this study, but could account for the increased fatigue and of pain in places other than the chest. No specific medication (other than steroids, routine analgesia) was given to alleviate these symptoms at the time the study was performed. However, the patient group over the study period received considerable comedication – a mean of 24, median of 17 different prescriptions, range 6–43.Cardiovascular agents were prescribed to 14 patients, the same number of steroids, 17 opiates and 6 patients broncodilators.

The lower scores on the role functioning scale at the end of the second cycle may be accounted for by increased levels of fatigue (Aaronson *et al*, 1993). There was also some indication that both cognitive and social functioning had declined, as well as global QoL. Declines in functioning were in the ‘moderate’ range according to Osoba's work, relating changes in QoL scores to patients' perceptions of the magnitude of change; the reduction in the global QoL score corresponded to a ‘little change’ (Osoba *et al*, 1998).

An alternative explanation for the reduction in some aspects of QoL is that these were not due to gemcitabine per se, but its administration at home. However, findings from the patient and family interviews suggest that this is unlikely to be the explanation. Patients and their carers were very positive about receiving chemotherapy at home rather than in the hospital: only two out of 12 thought it had any disadvantages, and all but one would recommend it to others in their situation. They reported feeling more relaxed and finding it less stressful. The fact that it took less time – an hour and a quarter compared to nearly 6 h for the hospital trip – was valued highly. Nevertheless, one in five, 13/16 of the patients included in the QoL analysis report declined to be interviewed or was too ill. This may have introduced some bias into the findings. Even if all three had found it a profoundly negative experience, this would not have changed the overall finding of the acceptability of the study – the majority of patients were very positive about receiving gemcitabine at home and preferred to it hospital care.

These findings are in agreement with previous studies that have investigated the acceptability of a home nursing oncology service in Australia (Lowenthal *et al*, 1996) and of a supportive home treatment programme for cancer patients with haemotological disorders (the majority of whom received blood and plasma transfusions) (Stockelberg *et al*, 1997), and of chemotherapy at home via continuous intravenous infusions (Sato *et al*, 1996). None of these studies explored the impact on patients' QoL using a prospective study design or validated measures. The median survival of 203 days (6.7 months) range 54–979 days was in line with the randomised trial of single-agent gemcitabine plus best supportive care *vs* best supportive care alone (Anderson *et al*, 2000).

Although patients may prefer domiciliary treatment, family members and others providing informal care to these patients may not. They may feel that it adds to the responsibilities that they carry, and may prefer that the patient receive hospital treatment. The importance of informal carers is increasingly recognised in oncology and, in particular, in palliative care. The inability of informal carers to continue to care due to exhaustion or to a lack of confidence leads to hospital admissions. Caring can impose a considerable load on informal carers, involving physical and emotional labour, and often restricting their social and working lives. Nevertheless, many report being glad to provide this care and see it as a natural extension of bonds of kin and affection. The Carers Act (1995) has stressed the importance of considering the needs of carers in their own right, rather than taking for granted that they will provide care when requested by the patient and/or by health and social services.

The impact of caring on the lives of informal carers did not increase between the baseline and the end of the second treatment cycle, nor did the levels of psychiatric morbidity, or subjective health status. Like the patients, the 17 informal carers who were interviewed at the end of the second cycle were overwhelmingly positive about the experience: they were pleased that the patients had not had to travel, wait at hospital and they had been more relaxed at home. The majority preferred home administration of gemcitabine, and would recommend it – rather than hospital treatment – to other families in the same situation.

From the available data, there does not appear to be any increased burden on health-care professionals due to the domiciliary administration of gemcitabine. Only three visits were made to patients by the domiciliary nurse where the purpose was to administer gemcitabine, but this was not possible. This has implications for planning such a service as do the distances travelled by the domiciliary nurse in order to administer the treatment. It should be noted that one centre was located at some distance from the domiciliary nurse base, and this has therefore increased the distances travelled by the nurses.

The results of this feasibility study suggest that transferring the administration of single-agent gemcitabine from the hospital to the patients' home (with effective nursing, medial support and clear communication) is acceptable to both patients and carers.
